# Cellular therapies in rheumatic and musculoskeletal diseases

**DOI:** 10.1016/j.jtauto.2024.100264

**Published:** 2024-12-16

**Authors:** Pedro Franco-Fuquen, Juana Figueroa-Aguirre, David A. Martínez, Eider F. Moreno-Cortes, Juan E. Garcia-Robledo, Fabio Vargas-Cely, Daniela A. Castro-Martínez, Mustafa Almaini, Januario E. Castro

**Affiliations:** a Division of Hematology and Medical Oncology, Mayo Clinic, Phoenix, AZ, USA; b Cancer Research and Cellular Therapies Laboratory, Mayo Clinic, Phoenix, AZ, USA; c Stanford University, Stanford, CA, USA; d Rheumatology, Allergy & Clinical Immunology Division, Mafraq Hospital, United Arab Emirates

**Keywords:** Adoptive immunotherapy, Chimeric antigen receptor, Cell- and tissue-based therapy, Autoimmune disease, Rheumatic and musculoskeletal disease

## Abstract

A substantial proportion of patients diagnosed with rheumatologic and musculoskeletal diseases (RMDs) exhibit resistance to conventional therapies or experience recurrent symptoms. These diseases, which include autoimmune disorders such as multiple sclerosis, rheumatoid arthritis, and systemic lupus erythematosus, are marked by the presence of autoreactive B cells that play a critical role in their pathogenesis. The persistence of these autoreactive B cells within lymphatic organs and inflamed tissues impairs the effectiveness of B-cell-depleting monoclonal antibodies like rituximab.

A promising therapeutic approach involves using T cells genetically engineered to express chimeric antigen receptors (CARs) that target specific antigens. This strategy has demonstrated efficacy in treating B-cell malignancies by achieving long-term depletion of malignant and normal B cells. Preliminary data from patients with RMDs, particularly those with lupus erythematosus and dermatomyositis, suggest that CAR T-cells targeting CD19 can induce rapid and sustained depletion of circulating B cells, leading to complete clinical and serological responses in cases that were previously unresponsive to conventional therapies.

This review will provide an overview of the current state of preclinical and clinical studies on the use of CAR T-cells and other cellular therapies for RMDs. Additionally, it will explore potential future applications of these innovative treatment modalities for managing patients with refractory and recurrent manifestations of these diseases.

## Graphical abstract

**Figure:**
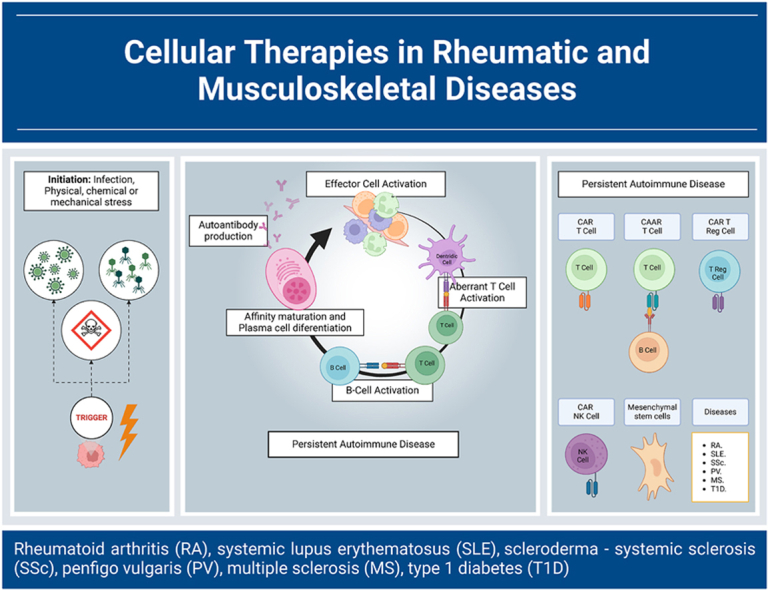

## 1 Introduction

Rheumatic and musculoskeletal diseases (RMDs) represent a heterogeneous group of more than 200 conditions affecting adults and children [1,2]. These diseases significantly impact the population's health and contribute to rising healthcare costs due to their chronic nature and the need for long-term treatments [3]. RMDs are characterized by degenerative, inflammatory, and autoimmune processes that result in pain, disability, diminished quality of life and life expectancy [[1], [2], [3]]. They are also associated with exacerbation of other chronic illnesses [4]. According to the 2010 World Health Organization (WHO) Global Burden of Disease Study, RMDs rank as the second leading cause of disability worldwide, following mental and behavioral disorders, with a prevalence exceeding 350,000 cases per million across all age groups [5]. Although significant progress in understanding and treating RMDs has been made, many patients experience persistent symptoms and disease-related complications [[1], [3]]. While treatments have evolved from early agents like salicylates and corticosteroids in the 1940s to modern synthetic and biological disease-modifying antirheumatic drugs (DMARDs), such as methotrexate and TNF inhibitors [6], RMDs are still incurable [3].

Chimeric antigen receptor (CAR) T-cell technology emerged in the late 1980s and was initially explored for its potential in treating HIV-infected patients [[7], [8], [9]]. However, the remarkable efficacy demonstrated by CAR T-cell therapies in hematological malignancies, including acute leukemia, aggressive lymphoma, and multiple myeloma, led to the expedited approval of six CAR T-cell products by the time of this review [[10], [11], [12], [13], [14]]. The fundamental mechanism underlying CAR T-cell therapy involves genetically modifying T cells to express chimeric receptors that combine an extracellular antigen-binding domain, typically derived from an antibody, with intracellular signaling domains that trigger activation of the immune effector cells (IEC) [15]. CAR T-cell specificity is achieved by the single chain fraction variable (scFv), which is an antibody-derived binding domain that enables the T cells or other IEC to redirect their cytotoxic activity towards specific target cells, independently of HLA antigen presentation and T-cell receptor (TCR) engagement [10,16].

CD19 CAR T-cell therapies in hematologic malignancies exhibit an on-target, off-tumor effect by targeting CD19 expressed on non-malignant B cells, resulting in B-cell aplasia. This prolonged B-cell aplasia serves as a surrogate marker for CD19 CAR T-cell activity and has been associated with improved prognosis and survival in patients with leukemia and lymphoma [[17], [18], [19]]. Because of these outcomes, there is growing interest in harnessing the power of CAR T-cell therapy for the treatment of rheumatic and musculoskeletal diseases (RMDs). Preliminary studies, including Phase I and II clinical trials, are evaluating the potential of leveraging CD19 CAR T-cell-induced B-cell aplasia to target autoreactive B cells, which play a central role in the pathogenesis and persistence of inflammatory processes within lymphoid tissues and affected organs (Table 1) [1,[20], [21], [22]]. These autoreactive B cells act as reservoirs of chronic disease, posing significant challenges to therapeutic strategies, even those utilizing B-cell-depleting monoclonal antibodies such as rituximab [[23], [24], [25]]. Preclinical studies have demonstrated the feasibility of CAR T-cell therapy in various RMDs, including rheumatoid arthritis (RA), Type 1 diabetes mellitus (T1D), systemic lupus erythematosus (SLE), and multiple [[26], [27], [28], [29]].

**Table 1:** FDA approved CAR T product Statistics.

Generic Name	Band Name	Target Antigen	Year of approval	Targeted Disease	Costimulatory Domain	Patient Population
Tisagenlecleucel	Kymriah	CD19	Aug/30/17	B-ALL	4-1BB, CD3-z	Children and young adults with R/R B-ALL.
				B-NHL		Adults with R/R B-NHL.
Axicabtagene ciloleucel	Yescarta	CD19	Oct/18/17	B-NHL	CD28, CD3-z	Adults with R/R B-NHL.
				FL		Adults with R/R FL.
Brexucabtagene autoleucel	Tecartus	CD19	Jun/24/20	ML	CD28, CD3-z	Adults with R/R MCL.
				B-ALL		Adults with R/R B-ALL.
Lisocabtagene maraleucel	Breyanzi	CD19	Feb/05/21	B-NHL	4-1BB, CD3-z	Adults with R/R B-NHL.
			Mar/14/24	CLL		
Ideacabtagene vicleucel	Abecma	BCMA	Mar/26/21	MM	4-1BB, CD3-z	Adults with R/R MM.
Ciltacabtagene autoleucel	Carvykti	BCMA	Feb/28/22	MM	4-1BB, CD3-z	Adults with R/R MM.

*Table footnote:***B-ALL=** B cell Acute Lymphoblastic leukemia; **B-NHL=** B cell non-Hodgkin Lymphoma; **R/R=**Relapse/Refractory; **FL=** Follicular Lymphoma; **MCL =** Mantle cell lymphoma; **MM** = Multiple Myeloma.

Additionally, a recent small-scale clinical trial demonstrated promising outcomes in the treatment of refractory SLE using anti-CD19 CAR T-cell therapy. Notable improvements were observed in disease activity, particularly renal involvement, laboratory parameters, symptomatology, and a reduced reliance on antirheumatic medications, leading to sustained remission without the need for ongoing pharmacological intervention [30]. However, it is important to recognize the potential safety concerns associated with CAR T-cell therapy, including immune effector cell-associated neurotoxicity syndrome (ICANS) and cytokine release syndrome (CRS) [31,32]. These adverse events can present with severe symptoms requiring intensive care management and, in some cases, have been associated with fatalities [10,33]. Therefore, further research is necessary to comprehensively assess the safety profile and therapeutic efficacy of cellular therapies in the management of rheumatic and musculoskeletal diseases (RMDs).

This review aims to provide a comprehensive overview of recent advances in the biology, preclinical, and clinical studies of cellular therapies for RMDs. Specifically, we summarize the latest literature on various cellular therapeutic modalities, including CAR-T cells, chimeric antigen receptor natural killer cells (CAR-NK cells), mesenchymal stem cells, and regulatory T cells (Tregs). These innovative therapies hold significant potential to revolutionize treatment strategies for patients with debilitating and refractory RMDs, with the ultimate goal of improving both quality of life and survival outcomes.

## 2 Historical milestones and development of cellular therapy technologies

Cellular therapy involves the use of living cells to restore or enhance the function of a patient's immune system. One of the most significant breakthroughs in this field was the development of CARs, which allow for the engineering of T cells to redirect their cytotoxic activity independently of human leukocyte antigen (HLA) presentation or T cell receptor engagement. The concept of CAR T-cells was first proposed by Zelig Eshhar, an Israeli scientist, in the late 1980s. His team's early experiments demonstrated that expressing an antibody recognition domain within T cells could specifically redirect cytotoxic activity, independent of the T-cell receptor or HLA antigen presentation [8,34].

Despite these promising early findings, it took nearly two decades to refine CAR designs to improve their safety and efficacy. In the early 2000s, the first clinical trials using CAR T cells were conducted in HIV-infected patients [9,[35], [36], [37], [38], [39], [40]]. These trials utilized lentiviral-mediated autologous T cell transduction with a CAR construct that included the extracellular and transmembrane domains of the CD4 molecule linked to the CD3-ζ signaling domain (CD4ζ CAR). The rationale for this approach was to exploit the chimeric CD4 receptor's ability to bind to HIV-infected T cells expressing the viral envelope protein, thereby facilitating the elimination of HIV-infected lymphocytes [41]. These pioneering studies, conducted in patients with both active viremia and chronic HIV-1 infection, demonstrated that the infusion of genetically modified T cells was feasible and safe [[35], [36], [37]]. A subsequent analysis conducted a decade later confirmed the long-term safety of retroviral-mediated T cell modification and demonstrated the persistence of CD4ζ CAR-modified T cells within the host immune system over an extended period [[41], [42], [43]].

Following these groundbreaking studies in HIV, the field of cellular therapy, particularly CAR T-cell technology, has experienced rapid and substantial progress. This progress underscores the immense therapeutic potential of harnessing the immune system to combat various diseases. With ongoing advancements in research and technology, cellular therapies are poised to revolutionize treatment approaches, offering novel strategies to improve patient outcomes. The following sections outline key milestones that led to the FDA approval of CAR T-cell therapies [[10], [14],^44^].

− 2010: The first successful clinical trial using CAR T-cells showed promise for treating chronic lymphocytic leukemia (CLL) [45].
− 2011–2012: Significant advancements were made in CAR design, including developing second-generation CARs with improved signaling domains.
− 2017: The U.S. Food and Drug Administration (FDA) approved the first CAR T-cell therapy, tisagenlecleucel (Kymriah), for pediatric acute lymphoblastic leukemia (ALL) [46].
− 2018: Another CAR T-cell therapy, axicabtagene ciloleucel (Yescarta), received FDA approval for certain types of aggressive non-Hodgkin lymphoma [10].
− 2020: Breyanzi (lisocabtagene maraleucel) became the third CAR T-cell therapy approved in the United States for certain types of non-Hodgkin lymphoma [14].
− 2021: Abecma (idecabtagene vicleucel) received FDA approval in March 2021 to treat relapsed or refractory multiple myeloma. This marked the first CAR T-cell therapy approved for multiple myeloma and the first against B cell maturation antigen (BCMA) [12].
− 2021: Tecartus (brexucabtagene autoleucel) received FDA approval In August 2021 for the treatment of mantle cell lymphoma in adults who have received at least one prior therapy [47].
− 2021: Tecartus (brexucabtagene autoleucel) received FDA approval in October 2021 for the treatment of relapsed or refractory B-cell precursor acute lymphoblastic leukemia in adults who have received at least one prior therapy [48].
− 2022: CARVYKTI (ciltacabtagene autoleucel) received FDA approval in February 2022 for the treatment of relapsed or refractory multiple myeloma in adults after four or more prior lines of therapy, including a proteasome inhibitor, an immunomodulatory agent, and an anti-CD38 monoclonal antibody [49].
− 2024: Breyanzi (lisocabtagene maraleucel) received FDA approval in March 2024 for the treatment of relapse or refractory chronic lymphocytic leykemua (CLL) and became the first CAR T-cell therapy approved for CLL therapy [50].

CAR T-cell constructs consist of four essential components: an extracellular antigen-binding domain, a hinge region, a transmembrane domain, and an intracellular signaling domain responsible for initiating T-cell activation [16,51]. The extracellular antigen-binding domain, primarily composed of the scFv, is critical for CAR T-cell function as it facilitates the recognition and redirection of T cells towards specific antigens. Derived from antibody sequences, the scFv includes the variable regions of both heavy and light chains linked by a short peptide sequence, forming a single polypeptide chain.

The hinge region connects the extracellular antigen-binding domain to the transmembrane domain, providing the flexibility needed to optimize antigen recognition. The transmembrane domain, a hydrophobic region that spans the CAR T-cell membrane, serves to anchor the CAR construct and ensure its stability within the cell membrane. It also facilitates signal transmission from the extracellular domain to the intracellular signaling components. Commonly used transmembrane domains are derived from molecules like CD8 and CD28, which play critical roles in T-cell function [16,20,52,53].

The intracellular signaling domain is essential for initiating and propagating signaling cascades upon antigen recognition. It consists of signaling motifs, such as CD3ζ or FcεRIγ, that activate downstream pathways involving protein kinases and transcription factors. Upon antigen binding, the CAR induces phosphorylation of immunoreceptor tyrosine-based activation motifs (ITAMs), which in turn recruit effector molecules, leading to T-cell activation, proliferation, and cytotoxic molecule release to eliminate target cells [16,20]. This mechanism closely parallels the activation process observed in unmodified T cells following antigen recognition via the T-cell receptor (TCR) [54].

**Fig. 1:**
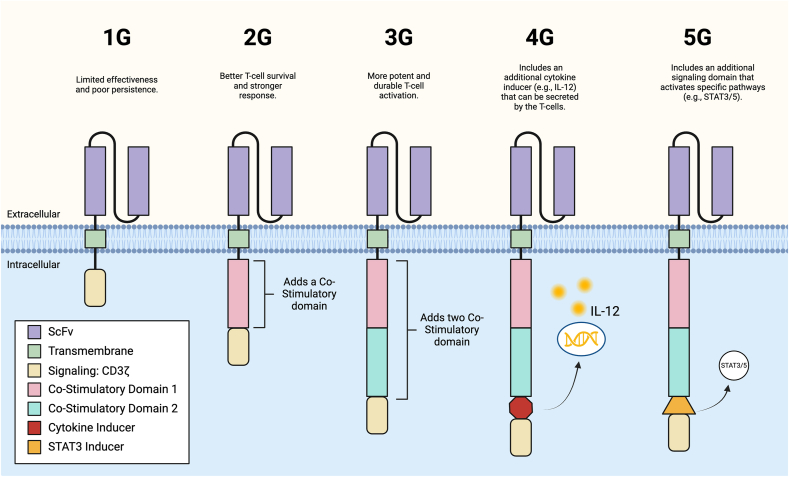
*Evolution of CAR T-Cell Generations.* Overview of CAR T-cell advancements from the first generation (1G) to the fifth generation (5G). Each generation introduces modifications to enhance T-cell activation, persistence, and specificity. Key additions include co-stimulatory domains, cytokine inducers, and signaling domains that improve T-cell responses in therapeutic applications.

Over time, CAR T-cell designs have undergone successive refinements across different generations to improve their safety and efficacy [51] (Fig. 1). First-generation (1G) CAR T-cells featured a single intracellular signaling domain, but suffered from high levels of tonic signaling and activation-induced cell death, which limited their persistence. To address these limitations, second-generation (2G) CAR T-cells were developed by incorporating an additional intracellular costimulatory domain (ICD), enhancing both activation and persistence [10,12,14,[55], [56], [57], [58], [59]]. Third-generation (3G) CAR T-cells introduced additional signaling domains, resulting in improved cytotoxicity, proliferation, and persistence [[59], [60], [61], [62], [63]].

Fourth-generation CAR T-cells, also known as armored CAR T-cells or T cells redirected for universal cytokine killing (TRUCKs), are engineered to secrete specific cytokines or immunomodulatory molecules upon antigen recognition [51,64,65]. This enhances the anti-tumor immune response by creating a localized inflammatory environment and recruiting additional immune cells to the tumor site. Most recently, fifth-generation or next-generation CAR T-cells have been engineered to activate alternative immune pathways, such as STAT3/5, further amplifying the anti-tumor immune response and enhancing therapeutic efficacy [66].

In addition to CAR T-cells, several alternative cellular therapies are being explored for cancer treatment, including:

**(1)** Tumor-infiltrating lymphocyte (TIL) therapy: This approach involves isolating, culturing, and expanding TILs from a patient's tumor for reinfusion. Encouraging results from melanoma trials have led to the FDA approval of TIL therapy in 2024 [67]. **(2)** Natural Killer (NK) cell therapy: NK cells, either alone or engineered to express CARs, have demonstrated cytotoxic activity against cancer cells and show promise in treating leukemia, lymphoma, and other malignancies. NK cells offer distinct advantages over T cells, including enhanced tumor penetration, broad cytotoxicity, and the ability to activate other immune cells [68,69]. **(3)** T-cell receptor (TCR)-based cellular therapy: This approach involves genetically modifying T cells to express TCRs that recognize tumor-specific antigens presented by HLA complexes. This strategy broadens the targeting spectrum to include intracellular antigens, expanding its potential application to solid tumors [[70], [71], [72]]. **(4)** Dendritic cell vaccines: Dendritic cells are isolated, loaded with tumor-specific antigens, and reinfused to elicit a multiparametric anti-tumor response, stimulating durable immune responses, including the activation of memory T cells [73,74]. **(5)** Targeting tumor-associated macrophages (TAMs): Depending on their polarization state (M1 vs. M2), TAMs can exert pro-tumor or anti-tumor effects. Research is focused on repolarizing TAMs toward an anti-tumor M1 phenotype and genetically modifying them to express CARs, thereby enhancing their cancer-fighting capabilities [69,75].

These innovative therapeutic approaches hold substantial promise, with many currently advancing through preclinical development and clinical trials. It is conceivable that some of these strategies could eventually be optimized for the treatment of rheumatic and musculoskeletal disorders (RMDs).

## 3 Understanding the mechanisms and progression of autoimmune diseases

The immune system is a highly sophisticated defense mechanism designed to recognize and eliminate pathogens such as viruses, bacteria, and abnormal cells [76,77]. It is tightly regulated by a complex network of signaling molecules and pathways, enabling rapid and precise responses to potential threats. However, when this balance is disrupted, the immune system may mistakenly target healthy tissues, leading to autoimmune diseases. These diseases occur when the immune system loses tolerance for the body's own cells and tissues [37] Autoimmune diseases are broadly classified into two categories: organ-specific and systemic. Organ-specific disorders, such as T1D and Hashimoto's thyroiditis, primarily target specific organs or tissues, whereas systemic conditions, such as SLE and RA, affect multiple organs and physiological systems. Each autoimmune disease is characterized by distinct mechanisms and symptoms, necessitating individualized diagnostic and treatment approaches [77,78].

**Fig. 2:**
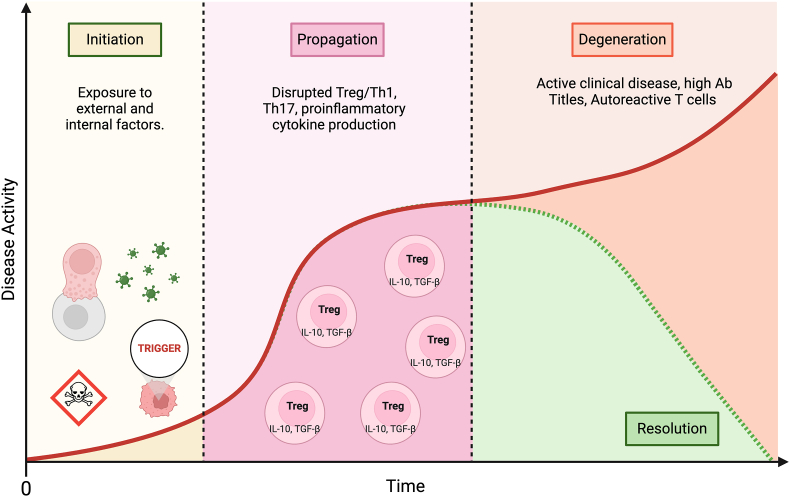
*Phases of Autoimmune Disease Progression.* The disease process begins with the Initiation phase, triggered by exposure to external and internal factors. This leads to Propagation, marked by disruptions in the balance of Treg, Th1, and Th17 cells and an increase in proinflammatory cytokine production. From there, the disease can progress in one of two directions: Resolution, where Treg-mediated suppression reduces disease activity, or Degeneration, characterized by active disease with high antibody titers and autoreactive T cells.

Despite their diversity, autoimmune diseases generally follow a three-phase progression: initiation, propagation, and resolution [77] (Fig. 2). The process begins with an **initiating event**, where the immune system mounts a response against the body's own antigens, typically triggered by genetic, environmental, or infectious factors. During the **propagation phase**, autoreactive T and B lymphocytes become activated and proliferate, producing autoantibodies and pro-inflammatory cytokines, which lead to tissue inflammation and damage. In an ideal scenario, regulatory mechanisms, such as regulatory T cells, suppress these autoreactive cells, **resolving** the inflammation and restoring immune tolerance. However, in many cases, the resolution phase fails, resulting in chronic inflammation and progressive tissue damage [77,78].

Understanding the stages of autoimmune disease progression is critical for developing effective diagnostic tools and personalized treatment strategies. Current research continues to explore the underlying mechanisms of autoimmune diseases, with the goal of improving therapeutic options and enhancing patient outcomes.

## 4 CAR-T cell strategies

Cell therapy for cancer patients aims to generate potent immune responses against specific tumor antigens, overcoming the limitations posed by tumor or tumor microenvironment (TME) immunosuppression [16,79]. Traditionally, anti-CD20 antibodies like rituximab have been used for B-cell depletion in conditions such as RA, multiple sclerosis, and vasculitis. However, responses in other rheumatic and musculoskeletal diseases (RMDs) remain inconsistent [1,[80], [81], [82], [83]]. This inconsistency may be attributed to the incomplete depletion of bone marrow-resident cells and the development of antibodies against the murine components of rituximab, compromising its efficacy and leading to adverse effects [84,85].

The success of CAR T-cell therapy in B-cell malignancies has paved the way for its potential application in RMDs [10,13,86]. CAR T-cell therapy has demonstrated significant clinical outcomes as a B-cell depletion strategy, as evidenced by the FDA's approval of the first CD19 CAR T-cell therapy in 2017 for acute lymphoblastic leukemia [87]. By 2024, six CAR T-cell products had received FDA approval for various B-cell malignancies [[10], [11], [12], [13], [14]] (Table 1). Efforts are focused on optimizing CAR constructs to enhance immune effector cell (IEC) functions, including cytotoxicity, persistence, and activity within the tumor microenvironment (TME) [16,[88], [89]]. A critical modifiable factor for improving CAR T-cell persistence is the selection and optimization of intracellular signaling domains (ICDs) [16,90,89]. Research into CAR molecular architecture aims to improve T-cell expansion, persistence, and clinical outcomes, particularly in solid tumors, where persistence is closely associated with therapeutic efficacy [[91], [92], [93]].

CAR T-cell therapy targeting CD19 presents a novel approach for modulating B-cell function in RMDs, offering potential advantages over traditional B-cell depletion methods [1,[20], [21], [22]]. The FDA's approval of inebilizumab for neuromyelitis optica spectrum disorder (NMOSD), coupled with promising results from studies using CD19-targeting CAR T-cells in SLE and refractory antisynthetase syndrome, supports this strategy [94]. Initial clinical data from seven patients with refractory SLE treated with CD19 CAR T-cells showed significant reductions in disease activity and autoantibody levels, with no relapses observed during long-term follow-up, indicating a favorable safety profile [95]. Phase 1 and 2 trials are underway to evaluate the use of CAR T-cells and other immune effector cells in autoimmune diseases, including RMDs. These trials aim to achieve durable immune cell depletion by targeting CD19, BCMA, or CD7, with ongoing studies showing promising results (NCT05239702) [96,97].

## 5 Chimeric autoantibody receptor-T (CAAR-T) cell strategies

CAAR-T cell therapy is a novel immunotherapy approach that differs from CAR-T cell therapy. While CAR-T cells target specific antigens on pathological cells, such as tumors, CAAR-T cells are designed to recognize the B cell receptor (BCR) expressed by self-reactive B cells. This approach utilizes an autoantigen as the binding domain on effector cells (e.g., T cells or NK cells) to bind the specific antigen recognition site of the BCR on autoreactive B cells. Therefore, CAAR-T cells can identify and eliminate pathogenic B cells involved in autoimmune processes without the need for HLA or TCR recognition [98,99].

**Fig. 3:**
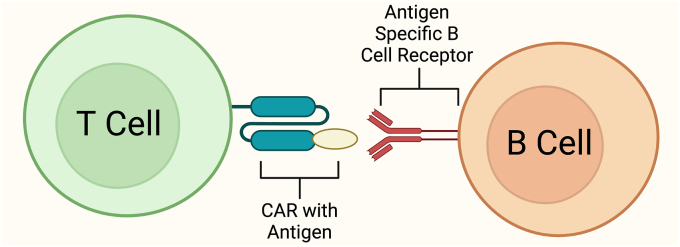
*Mechanism of CAAR T Cells in Autoimmune Disease.* Human T cells are engineered to express a Chimeric Autoantibody Receptor (CAAR) that targets autoreactive B cells producing cytotoxic autoantibodies, as seen in autoimmune diseases like Pemphigus vulgaris (PV). Upon binding to their target, CAAR T cells become activated, selectively depleting the autoreactive B cell population and thereby reducing disease activity by targeting the cells driving autoimmunity.

The CAAR construct consists of an extracellular domain coupled with transmembrane, co-stimulatory, and activation domains (Fig. 3). When CAAR-T cells bind to the autoantigen, they become activated and target autoreactive B cells for destruction. This strategy selectively depletes pathogenic B cell populations and shows great promise for treating a range of autoimmune diseases, offering a more tailored and potentially more effective approach compared to conventional CAR-T cell therapies [98,99].

Currently, two clinical trials are evaluating CAAR-T cell therapy targeting muscle-specific tyrosine kinase (MuSK) in myasthenia gravis and desmoglein-3 (Dsg-3) in mucosal-dominant pemphigus vulgaris (Table 2). Preclinical studies in a mouse model of pemphigus vulgaris (PV) have demonstrated that CAAR-T cells expressing the PV autoantigen Dsg-3 effectively deplete pathogenic B cells and induce long-term clinical improvements in PV symptoms [98,100].

**Table 2:** Overview of current clinical trials involving CAR T-cell Therapy in Autoimmune diseases.

Title	Sponsor	Condition/Disease	First Posted	NCT Number
A Study of CD19 Redirected Autologous T Cells for CD19 Positive Systemic Lupus Erythematosus (SLE)	Shanghai GeneChem Co., Ltd.	Systemic Lupus Erythematosus (SLE)	Jan/25/17	NCT03030976
Precision Diagnostics in Inflammatory Bowel Disease, Cellular Therapy and Transplantation (The PREDICT Trial) (PREDICT)	Boston Children's Hospital	Graft Vs. Host Disease	Dec/12/17	NCT03369353
		Inflammatory Bowel Diseases		
		Functional Gastrointestinal Disorders		
Treatment of Relapsed and/or Refractory AQP4-IgG Seropositive NMOSD by Tandem CAR T-cells Targeting CD19 and CD20	Chinese PLA General Hospital	Neuromyelitis Optica Spectrum Disorder	Jul/30/18	NCT03605238
Descartes-08 CAR-T Cells in Generalized Myasthenia Gravis (MG)	Cartesian Therapeutics	Myasthenia Gravis, Generalized	Oct/31/19	NCT04146051
Open-label Study to Determine the Maximum Tolerated Dose of DSG3-CAART in Mucosal-dominant PV Patients (mPV)	Cabaletta Bio	Mucosal -Dominant Pemphigus Vulgaris	Jun/09/20	NCT04422912
			
Safety and Efficacy of CT103A Cells for Relapsed/Refractory Antibody-associated Idiopathic Inflammatory Diseases of the Nervous System (CARTinNS)	Tongji Hospital	Autoimmune Diseases	Sep/23/20	NCT04561557
		Autoimmune Diseases of the Nervous System		
		Neuromyelitis Optica Spectrum Disorder		
		Myasthenia Gravis		
		Chronic Inflammatory Demyelinating Polyradiculoneuropathy		
		Immune-Mediated Necrotizing Myopathy		
A Study of CD19/BCMA Chimeric Antigen Receptor T Cells Therapy for Patients With Refractory Sjogren's Syndrome	Zhejiang University	Sjogren's Syndrome	Oct/20/21	NCT05085431
		Autoimmune Diseases		
A Study of CD19/BCMA Chimeric Antigen Receptor T Cells Therapy for Patients With Refractory Scleroderma	Zhejiang University	Scleroderma	Oct/20/21	NCT05085444
		Autoimmune Diseases		
A Study of CD19/BCMA Chimeric Antigen Receptor T Cells Therapy for Patients With Refractory Immune Nephritis	Zhejiang University	Immune Nephritis	Oct/20/21	NCT05085418
		Autoimmune Diseases		
		Lupus Nephritis		
A Study of CD19/BCMA Chimeric Antigen Receptor T Cells Therapy for Patients With Refractory Systemic Lupus Erythematosus	Zhejiang University	Systemic Lupus Erythematosus	Sep/01/21	NCT05030779
		Autoimmune Diseases		
Clinical Study of Targeting CD7 CAR-T Cells in the Treatment of Autoimmune Diseases	Zhejiang University	Crohn Disease	Feb/15/22	NCT05239702
		Ulcerative Colitis		
		Dermatomyositis		
		Still Disease		
		Autoimmune Diseases		
A Clinical Study of CD19/BCMA CAR-T Cells in the Treatment of Refractory POEMS Syndrome, Amyloidosis, Autoimmune Hemolytic Anemia, and Vasculitis	Zhejiang University	POEMS Syndrome	Mar/03/22	NCT05263817
		Amyloidosis		
		Autoimmune Hemolytic Anemia		
		Vasculitis		
A Study of Anti-CD19 Chimeric Antigen Receptor T-Cell (CD19 CAR T) Therapy in Subjects With Refractory Lupus Nephritis	Kyverna Therapeutics	Lupus Nephritis	Jul/10/23	NCT05938725
		Lupus Nephritis - World Health Organization (WHO) Class III		
		Lupus Nephritis - WHO Class IV		
Open-label Study to Evaluate the Safety of Various Dosing Regimens of MuSK-CAART for MuSK Myasthenia Gravis	Cabaletta Bio	MuSK Myasthenia Gravis	Jul/11/22	NCT05451212
CAR-T Cells Targeting Autoimmune Diseases	Shenzhen Geno-Immune Medical Institute	Autoimmune Diseases	Jul/15/22	NCT05459870
BCMA-CD19 cCAR T-cell Treatment of Relapsed/Refractory Systemic Lupus Erythematosus (SLE)	iCell Gene Therapeutics	Relapsed/Refractory Systemic Lupus Erythematosus (SLE)	Jul/26/22	NCT05474885
An Open-label Study to Assess Safety, Efficacy, and Cellular Kinetics of YTB323 in Severe, Refractory Systemic Lupus Erythematosus	Novartis Pharmaceuticals	Systemic Lupus Erythematosus (SLE)	Apr/04/23	NCT05798117
		Lupus Nephritis		
Evaluate the Safety and Efficacy of CAR-T Cells in the Treatment of R/R Neuromyelitis Optica	Zhejiang University	Neuromyelitis Optica	Apr/25/23	NCT05828212
Evaluate the Safety and Efficacy of CAR-T Cells in the Treatment of Refractory Myasthenia Gravis	Zhejiang University	Myasthenia Gravis	Apr/25/23	NCT05828225
Phase I Clinical Study of GC012F Injection in Treatment of Refractory Systemic Lupus Erythematosus	Zhejiang University	Systemic Lupus Erythematosus (SLE)	May/06/23	NCT05846347
			
Dual Target CAR-T Cell Treatment for Refractory Systemic Lupus Erythematosus (SLE) Patients	RenJi Hospital	Refractory Systemic Lupus Erythematosus (SLE)	May/15/23	NCT05858684
Universal CAR-T Cells (BRL-301) in Relapse or Refractory Autoimmune Diseases	Bioray Laboratories	Systemic Lupus Erythematosus (SLE)	May/16/23	NCT05859997
		Sjogren's Syndrome		
		Systemic Sclerosis		
		Inflammatory Myopathy		
		ANCA Associated Systemic Vasculitis		
		Antiphospholipid Syndrome		
A Study of CC-97540 in Participants With Severe, Refractory Systemic Lupus Erythematosus (SLE)	Juno Therapeutics, Inc., a Bristol-Myers Squibb Company	Systemic Lupus Erythematosus (SLE)	May/23/23	NCT05869955
			
Descartes-08 for Patients With Systemic Lupus Erythematosus (SLE-001)	Cartesian Therapeutics	Systemic Lupus Erythematosus (SLE)	Sep/14/23	NCT06038474
			
Safety and Efficacy of CD19 Targeted CAR-T Therapy for Refractory Autoimmune Disease	Chongqing Precision Biotech Co., Ltd	SLE (Systemic Lupus)	Sep/28/23	NCT06056921
		Sjogren's Syndrome		
		Systemic Scleroderma		
		Dermatomyositis		
		Anti-Neutrophil Cytoplasmic Antibody-Associated Vasculitis		
A Study of Anti-CD19 Chimeric Antigen Receptor T-Cell (CAR-T) Therapy in Subjects With Non-relapsing and Progressive Forms of Multiple Sclerosis	Stanford University	Multiple Sclerosis	Nov/18/23	NCT06138132
		Multiple Sclerosis, Primary Progressive		
		Multiple Sclerosis, Secondary Progressive		
CARTIMMUNE: Study of Patients With Autoimmune Diseases Receiving KYV-101 (CARTIMMUNE)	David Porter	Idiopathic Inflammatory Myopathies	Nov/30/23	NCT06152172
		Diffuse Cutaneous Systemic Sclerosis		
		SLE Nephritis		
		ANCA Associated Vasculitis		
A Study of Anti-CD19 Chimeric Antigen Receptor T-Cell Therapy in Subjects With Refractory Generalized Myasthenia Gravis	Kyverna Therapeutics	Myasthenia Gravis	Jan/05/24	NCT06193889
		Generalized Myasthenia Gravis		
CAR-T for Autoimmune Hemolytic Anemia Patients Who Have Failed Three or More Lines of Therapy	Institute of Hematology & Blood Diseases Hospital, China	Autoimmune Hemolytic Anemia	Jan/18/24	NCT06212154
		CD19 CAR-T Cell Infusion		
A Study to Evaluate the Safety, Tolerability, Efficacy, and Drug Levels of CC-97540 in Participants With Relapsing Forms of Multiple Sclerosis or Progressive Forms of Multiple Sclerosis	Juno Therapeutics, Inc., a Bristol-Myers Squibb Company	Multiple Sclerosis	Jan/23/24	NCT06220201
Study of Therapeutic Efficacy of Anti-CD19 CAR-T Cells in Children With Refractory Systemic Lupus Erythematosus	The Children's Hospital of Zhejiang University School of Medicine	Systemic Lupus Erythematosus	Jan/25/24	NCT06222853
		CAR-T Cell Therapy		
CNCT19 for Patients With Autoimmune Hemolytic Anemia After Failure ≥3 Lines of Therapy	Institute of Hematology & Blood Diseases Hospital, China	Autoimmune Hemolytic Anemia	Jan/30/24	NCT06231368
		Autologous CD19 CAR-T		
		Failure of Three or More Lines of Therapy		
A Study of C-CAR168 in the Treatment of Autoimmune Diseases Refractory to Standard Therapy (CAR-AID)	RenJi Hospital	Systemic Lupus Erythematosus (SLE)	Feb/08/24	NCT06249438
		Immune-Mediated Necrotizing Myopathy		
		Neuromyelitis Optica Spectrum Disorders		
		Multiple Sclerosis-Relapsing-Remitting		

## 6 CAR-T-REG cell strategies

Tregs engineered to express CARs represent a novel immunotherapy approach for treating autoimmune diseases [101]. Unlike CAR-T and CAAR-T therapies, which target pathogenic cells or autoreactive B cells, CAR-Treg therapy leverages the immunosuppressive function of Tregs to restore immune balance [[102], [103], [104]].

Tregs are a specialized subset of T cells critical for maintaining self-tolerance and regulating immune responses in various conditions, including cancer, chronic inflammation, and infectious diseases. These cells migrate to inflamed tissues, where they reduce cellular activation, inflammation, and overall immune responses [105]. Tregs exhibit tissue-specific characteristics and express different molecules in varying ratios, depending on the disease context. For example, in SLE, a reduction in anti-inflammatory molecules such as IL-10 and CTLA-4 is attributed to decreased Treg numbers, while in Crohn's disease, impaired Treg function causes similar reductions. Understanding these variations is essential when designing Treg-based therapies for autoimmune diseases [[106], [107], [108], [109]].

CAR-Treg therapy involves genetically modifying Tregs to express CARs that recognize antigens associated with autoimmune diseases. These engineered CAR-Tregs migrate to sites of inflammation and suppress self-reactive immune cells, aiming to mitigate and control the pro-inflammatory effects of autoimmune diseases [103].

CAR-Treg therapy has progressed beyond preclinical studies and is now being evaluated in phase 1 and 2 clinical trials. Examples include CAR-Treg therapy for living donor renal transplant recipients (STEADFAST, NCT04817774), and CAR-Tregs targeting HLA-A2 MHC complexes, CD19 B cells, insulin, HiP2, GAD65 beta-cell epitopes, citrullinated vimentin (CV), and myelin oligodendrocyte glycoprotein (MOG), among others [110,111]. This targeted, tolerogenic therapeutic approach aims to minimize the risks of broad immunosuppression and the associated complications often seen with conventional therapies. Ongoing research and clinical trials are exploring the potential of CAR-Treg therapy across various autoimmune conditions to improve disease outcomes while minimizing adverse effects [110,112,113].

## 7 NK cells

NK cells cells are cytotoxic lymphocytes that play a critical role in the innate immune system, providing early defense against infections and cancer [114]. Unlike other immune cells, NK cells can recognize and eliminate infected or abnormal cells without prior sensitization or the need for specific antigen recognition [115]. NK cells exert their effects by releasing cytotoxic granules containing perforin and granzyme, inducing apoptosis through death receptors, and engaging in antibody-dependent cellular cytotoxicity (ADCC) [114,[116], [117], [118]].

In autoimmune diseases, which are primarily driven by dysregulated T and B lymphocytes, NK cells have been implicated in the pathogenesis of rheumatic and RMDs [115,119,120]. Altered NK cell functions, such as impaired cytotoxic activity, have been observed in conditions such as T1D [[121], [122], [123]], RA [[124], [125], [126], [127]], and SLE [128]. Reductions in peripheral blood NK cell counts in autoimmune patients may reflect their migration to inflamed tissues, which can influence immune responses [114]. Additionally, distinct NK cell subsets, characterized by differential CD56 expression, have been identified as potential markers for disease activity in systemic sclerosis.

NK cells are emerging as therapeutic targets in autoimmune diseases due to their involvement in disease progression, which is modulated by specific activating or inhibitory receptors. Modifying NK cell activity or targeting specific NK cell subsets offers innovative treatment approaches. Preclinical studies have shown that adoptive transfer of NK cells can reduce disease severity in animal models of autoimmune diseases [114,115,129].

Innovative strategies have also been explored to redirect NK cell cytotoxicity toward follicular helper T (Tfh) cells, which play a key role in autoantibody production by promoting aberrant B cell activation and differentiation within germinal centers. Elevated circulating Tfh cell numbers have been correlated with increased autoantibody titers in diseases like SLE, underscoring the pivotal role of Tfh cells in autoimmune pathology. Experimental models further demonstrate the necessity of Tfh cells for the development of systemic autoimmunity, highlighting their potential as therapeutic targets [[130], [131], [132], [133], [134], [135], [136]].

New therapeutic strategies utilizing programmed cell death protein 1 (PD-1) expression on Tfh cells have emerged, offering a targeted approach through CAR-NK cells [130]. his novel method employs a CAR construct designed to recognize PD-1 via a PD-L1 extracellular domain, enabling the selective elimination of Tfh cells with high PD-1 expression while sparing other T cell subsets. This precise targeting minimizes off-target effects and enhances therapeutic efficacy, particularly in autoimmune diseases where dysregulated Tfh responses are central to disease pathology [130].

In the context of CAR therapy, NK cells offer several advantages over T cells, including γδ T cells. NK cells have a more favorable safety profile, with reduced risks of severe adverse effects such as CRS and neurotoxicity, making them a safer option for immunotherapy [137]. Additionally, NK cells can be derived from various sources, including cord blood, stem cells, or cell lines, allowing for the development of "off-the-shelf" CAR-NK cell therapies [138]. This approach reduces manufacturing costs and enhances the accessibility of the therapy. Early trials have demonstrated that CAR-NK cells exhibit minimal adverse effects and lower toxicity, positioning them as a promising alternative to CAR-T cells, further supporting the growing role of cellular therapies in the treatment of autoimmune diseases [127,130].

## 8 Mesenchymal stem cell applications

Mesenchymal stem cells (MSCs) possess unique biological characteristics that make them highly promising for therapeutic applications. Their multipotency enables differentiation into various cell lineages, including bone, fat, and cartilage, while their robust self-renewal capacity ensures a sustained source of viable cells. These attributes make MSCs particularly attractive for tissue repair and regeneration strategies [139,140].

In addition to their regenerative capabilities, MSCs exhibit significant immunomodulatory properties, allowing them to modulate immune cell functions, including those of cytotoxic T cells and Tregs [141]. This immunomodulatory capacity contributes to their anti-inflammatory effects, positioning MSCs as promising candidates for cell-based therapies in autoimmune diseases such as RA, where inflammation and joint damage are central features [142].

MSCs exert potent immunomodulatory effects by suppressing pro-inflammatory immune cells and promoting Treg expansion, thereby downregulating inflammatory processes [[143], [144], [145]]. Their low immunogenicity allows for allogeneic transplantation, alleviating concerns about donor compatibility [146,147]. Additionally, MSCs offer exciting potential for combination therapy with existing RA medications, potentially providing synergistic therapeutic benefits. Clinical trials have demonstrated encouraging safety profiles, with minimal adverse effects reported in MSC-based therapies [148].

Although further research and clinical trials are needed to optimize their therapeutic use, MSCs offer hope for RA patients, presenting a promising avenue for combating this debilitating disease. Current MSC-based therapy protocols in RA focus on modulating immune responses and reducing arthritis-associated inflammation [141]. These protocols have shown consistent therapeutic benefits in preclinical studies, despite variations in tissue sourcing, MHC contexts, routes of administration, and animal models. The optimal timing for MSC infusion appears to be during the early phases of inflammation, although long-term efficacy remains to be fully investigated [149].

Early clinical data on MSC-based therapy for RA have shown promising results. A phase I/II trial conducted in China by Wang et al. demonstrated long-term beneficial effects when MSCs were combined with low doses of disease-modifying antirheumatic drugs (DMARDs) in 172 patients with active RA who had inadequate responses to conventional medication [150,151]. Another study in patients with refractory RA who had failed multiple biological treatments reported that intravenous infusions of allogeneic expanded adipose-derived MSCs (Cx611) were generally well-tolerated, with transient fever being the most common adverse reaction. The study also demonstrated a favorable response according to European League Against Rheumatism (EULAR) criteria, highlighting the potential therapeutic effects of MSC administration in refractory RA patients [152].

Exploring modified MSCs is another promising avenue, with preclinical studies showing potential benefits from genetically or otherwise modified cells [153]. The future of MSC therapy for RA and other RMDs promises significant advancements, guided by preclinical findings and emerging trends [[154], [155], [156], [157], [158]]. Notably, there is a growing recognition that early intervention may be key. Preclinical data suggest that the initial stages of inflammation provide an optimal window for MSC infusion, potentially maximizing treatment efficacy. Future trials will likely prioritize recruiting RA patients during these early phases [153].

The future of MSC therapy for RA and other RMDs will depend on advancements in preclinical studies, exploration of modified MSCs, assessment of long-term efficacy, optimization of products and protocols, and the identification of biomarkers predictive of patient response. These developments aim to unlock the full potential of this therapy, offering hope to patients with unmet medical needs in the fight against RMDsa [153].

## 9 Specific application of CAR-T cells in human autoimmune rheumatic diseases

### 9.1 CAR-T cells in rheumatoid arthritis (RA)

Advancements in RA research have provided significant insights into its pathogenesis, particularly emphasizing the pivotal roles of T and B cells in the dysfunctional immune microenvironment. These discoveries have led to innovative therapeutic approaches, such as rituximab, which effectively reduces autoantibody levels by targeting B cells [159]. Building on these breakthroughs, preclinical studies have identified specific antigens associated with RA pathogenesis that could be targeted using CAR-T cell therapy.

CAR-T cells can be engineered to express receptors that recognize B cell receptors (BCRs) on autoreactive B cells. This approach has already been successfully demonstrated in PV, where autoreactive B cells expressing anti-Dsg-3 BCRs were eliminated by targeting the specific autoantigen, Dsg-3. A similar strategy could be applied to RA treatment [160]. Zhang and colleagues have proposed an innovative method utilizing multiple citrullinated peptide epitopes, which are strongly associated with RA pathogenesis and serve as potential therapeutic targets.

Autoreactive B cells bind specifically to citrullinated protein epitopes, contributing to disease development and progression [161]. By employing anti-fluorescein isothiocyanate (FITC) labeled immunodominant peptides, Zhang and colleagues were able to selectively target autoreactive B cells expressing BCRs that recognize citrullinated protein epitopes [26]. FITC-labeled peptides bind to these autoreactive B cells, enabling anti-FITC CAR-T cells to redirect and eliminate the targeted B cell populations. This strategy offers a precise and customized approach to target autoreactive B cells in RA, potentially reducing disease activity and inflammation.

The CAR-T cell universal system allows for a personalized strategy that targets multiple BCRs, eliminating different subsets of autoreactive B cells that recognize citrullinated protein epitopes. This tailored approach minimizes the risk of broad B cell depletion seen with anti-CD20 therapy, allowing treatment to be adjusted according to each patient's unique profile [26].

While these studies show great promise, it is essential to recognize that translating CAR-T cell therapy from preclinical research to clinical practice requires thorough evaluation and validation. Additionally, optimizing the selection of target antigens and further understanding the complex interactions within the RA immune microenvironment will be crucial for the successful application of CAR-T cell therapy in RA.

### 9.2 CAR-T cells in systemic lupus erythematosus (SLE)

SLE is a complex autoimmune disorder characterized by abnormal immune responses against self-antigens, resulting in widespread inflammation and damage to multiple organs. Dysregulation of both the innate and adaptive immune systems, involving T cells, B cells, and antigen-presenting cells (APCs), plays a central role in the pathogenesis of SLE [162]. While T cells contribute to the breakdown of self-tolerance to multiple self-antigens, the clinical manifestations of SLE primarily result from autoantibodies, leading to type II and III hypersensitivity reactions [163]. These autoantibodies contribute to various symptoms, including cutaneous lupus [164], lupus nephritis [165], neuropsychiatric lupus [166], and lupus-associated serositis [167]. Consequently, therapeutic strategies for SLE predominantly target immune cells, mainly B or plasma cells [168,169]. Several anti-B cell therapies, such as rituximab and belimumab, have been employed, with different degrees of clinical efficacy [169].

CARs are engineered molecules that redirect the specificity of transduced cells towards specific antigenic targets [170]. While CARs have been extensively studied in oncology, particularly in targeting the B-cell surface antigens CD19 and BCMA, their application in autoimmune diseases like SLE is still emerging [30]. Autoreactive B cells in SLE have long been considered therapeutic targets, and CAR-T cell strategies targeting CD19 may achieve more effective and durable B-cell depletion, particularly in inflamed tissues affected by SLE [30,171].

Preclinical studies have demonstrated that CAR-T cells targeting BCMA or CD19 can effectively deplete autoreactive B cells, alleviating disease symptoms in lupus-prone mouse models [172,173]. These studies reported significant reductions in anti-nuclear autoantibody (ANA) titers, serum autoantibody levels, proteinuria, and renal pathology following CAR-T cell treatment [172,173]. Initial clinical applications of anti-CD19 CAR-T cells in patients with refractory SLE have shown rapid clinical remission, sustained B-cell depletion, and swift elimination of serum anti-double-stranded DNA antibodies, with manageable adverse effects [94].

A recent case series have further demonstrated the potential of CD19-targeted CAR-T cells in inducing remission in patients with refractory SLE [174]. The study reported a cohort of patients achieving disease remission with significant reductions in SLE Disease Activity Index (SLEDAI) scores and normalization of complement factor levels after CD19 CAR-T cell treatment. B-cell reconstitution was observed after a median of 112 days, but importantly, patients continued to experience remission for up to 29 months post-treatment. This reconstitution was characterized by a predominantly naive, non-class-switched B-cell phenotype, with a notable absence of disease-associated memory B cells, suggesting that CD19 CAR-T cell therapy may have induced an "immune reset" rather than a simple B-cell depletion [30,95,174].

While CAR-T cell therapy has achieved notable success in hematological malignancies, its application in autoimmune diseases like SLE remains in the early stages. Ongoing clinical trials are investigating the safety, efficacy, and long-term effects of CAR-T cell therapies in SLE. Despite promising initial data, several questions remain unanswered, including the durability of responses after B-cell repopulation and the identification of the optimal patient population that will benefit most from this treatment.

### 9.3 CAR-T cells in scleroderma - systemic sclerosis (SSc)

Scleroderma is an autoimmune disorder characterized by skin fibrosis and constriction, which can overlap with systemic sclerosis (SSc) when fibrosis extends to the lungs or other visceral organs [175,176]. In these conditions, elevated CD19 expression in B cells may contribute to the activation and hyperresponsiveness of memory B cells, which are associated with autoantibody production and fibrosis.

While rituximab has demonstrated efficacy in some SSc patients, the pathophysiology of the disease implicates not only mature B cells but also B-cell precursors and plasmablasts, which express lower levels of CD20 [177]. As a result, anti-CD20 agents may not fully target these cell populations, whereas anti-CD19 therapies could offer more comprehensive efficacy [178]. For instance, autologous stem cell transplantation has shown significant clinical benefit by inducing deeper global B-cell ablation [179]. However, this procedure is associated with high morbidity and mortality rates, especially in patients with advanced disease. Therefore, anti-B cell therapies, including CD19 or BCMA CAR-T cell therapy, present a promising therapeutic approach for treating SSc.

In May 2023, the first case report of an SSc patient treated with CD19 CAR-T cell therapy was published. The patient, a 60-year-old man with diffuse SSc and advanced cardiac and pulmonary involvement, had not responded to methotrexate and mycophenolate. Following the CD19 CAR-T cell infusion, complete B-cell depletion was achieved by day 7, accompanied by mild fever (CRS grade I) without neurotoxicity. Subsequent follow-up revealed seroconversion in antibody titers for antinuclear antibodies (ANAs) and anti-RP11 (subunit of RNA polymerase III), along with improvements in cardiac, cutaneous, and joint manifestations, while pulmonary function remained stable. Although the treatment showed effectiveness in these areas, lung pathology and function tests did not improve, consistent with the typically irreversible nature of pulmonary involvement in scleroderma [177].

Additionally, an ongoing phase I trial in China is investigating the safety and efficacy of CD19 CAR-T cell therapy in SSc patients (NCT05085444). To date, three patients have undergone lymphodepletion chemotherapy with fludarabine and cyclophosphamide before receiving infusions of 1-3x10^6^/kg CD19^+^ CAR-T cells. Monitoring of CAR-T cells and CD19^+^ B cell levels in peripheral blood post-infusion revealed detectable CAR-T cells in the first patient nine months after infusion, accompanied by clinical improvement, particularly in interstitial lung fibrosis. Reports on the clinical effectiveness of the remaining two patients are pending, but the therapy has been well-tolerated with no reported adverse events (NCT05085444).

### 9.4 CAAR-T cells in PENFIGO vulgaris (PV)

PV is a severe autoimmune blistering disorder caused by autoantibodies targeting desmoglein-1 and -3 (Dsg1, Dsg3), essential proteins for keratinocyte adhesion [180]. PV primarily affects the skin and mucous membranes, with mortality rates approaching 10 % [181]. Disruption of Dsg by autoantibodies leads to widespread blister formation, increasing the risk of septicemia, a leading cause of mortality.

The standard treatment for PV involves systemic corticosteroids and immunosuppressive agents such as azathioprine and mycophenolate mofetil. In severe cases, intravenous corticosteroid pulse therapy and CD20 depletion with rituximab have been used. However, these treatments typically induce only short-term remission, with most patients experiencing relapses [182]. Given the significant adverse effects of long-term immunosuppression and the limited durability of these treatments, there is a clear need for improved, targeted therapies.

PV presents a well-defined antigenic target, unlike many systemic autoimmune diseases, making it feasible to develop therapies that specifically target autoreactive B cells. CAAR T cells have emerged as a promising strategy, selectively targeting anti-Dsg3 BCR + B cells while minimizing the risks of generalized immunosuppression [183].

In one study, Dsg3 CAAR-T cells were engineered using second-generation CAR-T constructs similar to those used in hematologic malignancies [184]. These cells combined the Dsg3 autoantigen with a CD28 transmembrane domain and a CD137-CD3ζ signaling domain. Preclinical data demonstrated that Dsg3 CAAR-T cells selectively targeted anti-Dsg3 BCR + cells while sparing BCR-cells, showing no interaction with soluble serum anti-Dsg3 IgG. The therapy significantly reduced Dsg3 serum antibody levels and controlled disease progression in PV models without off-target cytotoxicity in keratinocytes or human skin xenograft mouse models, indicating a favorable safety profile.

Further studies by Lee et al. confirmed these findings, showing specific depletion of Dsg-3 BCR + cells using primary anti-Dsg-3 B cells and improved blistering in a mouse model. The activity of CAAR-T cells was dose-dependent, with no evidence of off-target interactions [100]. This robust preclinical data led to the initiation of the first human Phase 1 clinical trial evaluating Dsg3 CAAR-T cells (NCT04422912 – Table 2). While the results from human trials are still pending, these innovative therapies hold promise for more effective and potentially curative treatments for PV.

### 9.5 CAR-T cells in multiple sclerosis (MS)

MS is a complex cell-mediated autoimmune disorder affecting the central nervous system (CNS), characterized by demyelination, neuroinflammation, and axonal damage. The disease manifests through a variety of neurological symptoms such as optic neuritis, partial myelitis, focal sensory disturbances, and brainstem syndromes [185]. While the specific autoantigens involved in MS pathogenesis remain unclear, evidence suggests that CD4^+^ and CD8^+^ T cells mediate autoimmune responses against CNS myelin antigens, particularly involving Th1 and Th17 CD8^+^ myelin-reactive T cells [186].

Current therapeutic strategies for MS primarily focus on disease-modifying therapies (DMTs), which are administered either orally or intravenously, including monoclonal antibodies (mAbs). These treatments reduce annual relapse rates by 29%–68 %, but they often fail to prevent disease progression and carry an increased risk of infection186. Targeting CD20^+^ B cells with mAbs has shown clinical benefits in MS, and CAR-T cell-mediated B cell depletion has been explored as a potential therapeutic approach, demonstrating efficacy in both peripheral tissues and the CNS in preclinical models [187].

The adoptive transfer of CAR-Treg cells has emerged as a leading strategy for CAR-based treatments in MS [188]. Research in the experimental autoimmune encephalomyelitis (EAE) mouse model has led to significant advances in this area, where CAR-Tregs targeting CNS antigens, such as myelin oligodendrocyte glycoprotein (MOG) and proteolipid protein (PLP), have demonstrated both preventative and therapeutic effects [189]. In particular, Fransson et al. utilized a lentiviral vector system to engineer CD4^+^ T cells expressing a CAR targeting MOG and murine FoxP3 for differentiation into Tregs. Their in vitro and in vivo studies demonstrated that these CARαMOG-FoxP3-Tregs effectively suppressed T cell proliferation in the presence of MOG + cells and activated macrophages30. Similarly, bi-specific CAR strategies, such as those targeting both MOG and neurofilament medium (NF-MT), have shown promise in reducing EAE severity in C57BL/6 mice, further supporting the potential of CAR-Treg therapy in human MS [189,190].

Recent work has emphasized the potential for antigen-specific Treg therapies in MS treatment. These therapies can be designed to enhance immune suppression while simultaneously promoting tissue repair at sites of CNS inflammation. For example, engineered Tregs expressing antigen-specific chimeric receptors can modulate neuroinflammation and facilitate remyelination by promoting oligodendrocyte differentiation [191]. The ability of Tregs to exert local tissue repair in CNS lesions, a critical aspect of MS pathology, represents a major advancement in targeted cellular therapies193.

While CAR-Treg-based therapies for MS are still in the preclinical stage, the promising results observed in EAE models suggest that these therapies could provide long-lasting disease suppression with minimal off-target effects. Ongoing clinical studies, such as those investigating the administration of autologous Tregs in patients with relapsing-remitting MS (RRMS), will help determine the translational potential of this approach [191]. However, several challenges remain, including the need for further optimization of CAR constructs to improve the persistence and functionality of the engineered Tregs within the inflammatory milieu of the CNS [185,[189], [190], [191]].

Ultimately, the success of CAR-T cell therapy in MS will depend on achieving a balance between effective immune suppression and avoiding long-term systemic immunosuppression. Future studies are likely to focus on refining CAR design to enhance Treg stability and specificity, as well as identifying optimal antigenic targets to achieve sustained therapeutic benefits.

### 9.6 CAR-T cells in type 1 diabetes (T1D)

T1D arises from the activation of autoreactive T cells that inappropriately target and destroy pancreatic β-cells, resulting in insulin deficiency and subsequent hyperglycemia. Despite extensive research into the autoimmune mechanisms underlying T1D, translating these insights into effective therapeutic strategies for disease prevention has remained challenging [185]. A major focus of current T1D research is the development of antigen-specific therapies. While numerous islet antigens could potentially serve as targets for inducing tolerance, evidence suggests that T cells recognizing epitopes within (prepro)insulin play a pivotal role in islet autoimmunity, making (prepro)insulin a prime candidate for antigen-specific interventions [185].

Zhang and colleagues engineered a second-generation CAR-T cell incorporating a CD28 co-stimulatory domain to target the IAg7-B:9–23(R3) complex using the mAb287 monoclonal antibody [186]. In the NOD mouse model of spontaneous diabetes, the B:9–23 peptide of the insulin B chain contains at least one critical epitope; notably, mice expressing insulin molecules with a single amino acid mutation (alanine instead of tyrosine at position B16) are fully protected from the disease [187].

Kappler and colleagues demonstrated that the majority of islet-infiltrating B:9-23-specific CD4^+^ T cells in NOD mice recognize complexes in which the peptide is bound in the energetically unfavorable "register 3," positioning the positively charged Arg22 in the basic pocket 9^189,190^. To specifically target the IAg7-B:9–23(R3) complex, the mAb287 monoclonal antibody was developed, which selectively binds this complex without recognizing the free peptide or other peptide-bound complexes [186]. Using this antibody, a CAR construct was designed to develop CAR-T cells [186]. These CAR-T cells effectively eliminated APCs expressing the IAg7-B:9–23(R3) complex in vitro and delayed the onset of T1D in preclinical models. However, the protective effect was not permanent. Despite this, targeting APCs presenting pathogenic MHC class II peptide complexes with CAR-T cells remains a promising approach for treating T1D and related autoimmune disorders [27].

Efforts to generate a well-defined, consistently functional antigen-specific CAR-Treg population have also gained attention as a therapeutic strategy. Developing CAR-Tregs for T1D treatment offers promise due to their off-the-shelf availability and customizable design, minimizing off-target systemic suppression [190]. An insulin-specific autoantigen, prominent in T1D, was selected to develop a CAR-Treg strategy [191]. Using a phage display library, scFvs with the highest insulin-binding affinity were identified [192].

T-cell specificity was redirected toward insulin through CAR technology, and effector T cells (Teff) were transduced into Tregs via Foxp3 transduction. CD4^+^ Teff cells were transduced with a CD28.CD3ζ second-generation CAR construct containing insulin-specific scFvs and a Foxp3 sequence, reprogramming CD4^+^ T cells into insulin-specific Tregs (CAR-cTregs) [192]. Importantly, CAR-cTregs exhibited the same phenotype and function as natural Tregs, proliferating in the presence of insulin and suppressing the proliferation of allospecific CD8^+^ Teff cells. Although these CAR-cTregs could not halt diabetes progression in NOD female mice, they remained detectable up to 17 weeks post-adoptive transfer, comprising 2–4% of all splenic Tregs [192].

## 10 Practical considerations for cellular therapy in autoimmune diseases

The clinical implementation of CAR T-cell therapy in autoimmune diseases requires careful consideration of several practical factors to optimize outcomes and mitigate risks. These include selection criteria, patient follow-up protocols, management of adverse events, and monitoring for secondary malignancies and infections.

### 10.1 A)patient selection and exclusion criteria for car T-cell therapy

#### 10.1.1 Selection criteria

1.1) **Disease Severity and Refractoriness:** Eligible candidates must present with refractory or relapsed disease that has failed at least two lines of standard therapy, including immunosuppressive agents or biologics. For instance, anti-CD19 CAR T-cell therapy has demonstrated success in SLE, effectively targeting the autoreactive B cells that drive disease progression. This therapeutic approach has been pivotal for patients who have exhausted conventional treatment options [193].
1.2) **Target Antigen Expression:** A critical prerequisite for CAR T-cell therapy is confirming the presence of the target antigen to ensure specificity and efficacy. In B-cell-mediated autoimmune disorders such as SLE or idiopathic inflammatory myositis, CD19 expression on B cells can be validated through flow cytometry or immunohistochemistry. This verification minimizes the risk of off-target effects while optimizing therapeutic precision [193,194].
1.3) **Age and Performance Status:** Patients should exhibit sufficient functional reserve to tolerate therapy. An Eastern Cooperative Oncology Group (ECOG) performance status of ≤2 or a Karnofsky Performance Scale score of ≥70 % is generally required. While there are no universal age limits, pediatric and geriatric patients are evaluated on an individual basis, taking into account their overall health and the presence of comorbidities [195].
1.4) **Baseline Immune Status:** Adequate immune competence is essential for successful CAR T-cell manufacturing and post-treatment efficacy. Patients must exhibit sufficient levels of functional T cells, as deficiencies in key T-cell subsets may compromise the effectiveness of therapy. Immunophenotyping is often employed to identify potential immune deficits that could impact outcomes [193,196].
1.5) **Organ Function:** Baseline organ function must meet specific thresholds to reduce the risk of therapy-related toxicities. Hepatic function should include a serum bilirubin level of <1.5 × the upper limit of normal (ULN) and transaminases (AST and ALT) < 2.5 × ULN. Renal function should reflect a creatinine clearance >60 mL/min, and cardiac function must demonstrate a left ventricular ejection fraction ≥50 %. These parameters help ensure that patients can safely undergo treatment without undue risk of organ-related complications [195].

#### 10.1.2 Exclusion criteria

Exclusion criteria for CAR T-cell therapy are designed to minimize the risk of complications, including severe toxicities and treatment failure. Patients with active infections, whether bacterial, viral, or fungal, are ineligible until these infections are resolved or effectively managed [197]. Reactivations of latent infections, such as hepatitis B or C and cytomegalovirus, represent significant contraindications unless prophylactic antiviral therapy has been initiated [193]. Individuals with severe comorbidities are also excluded to prevent adverse events that could arise during treatment. For instance, conditions like severe heart failure, recent myocardial infarction, or uncontrolled arrhythmias increase cardiovascular risk, while severe obstructive or restrictive pulmonary diseases heighten the likelihood of respiratory complications [[198], [199]]. Similarly, hepatic dysfunction, including cirrhosis or advanced liver impairment classified as Child-Pugh class C, can exacerbate therapy-related toxicity and precludes safe administration [[200], [201]].

Pre-existing neurological conditions such as epilepsy, prior strokes, or progressive neurodegenerative diseases also render patients unsuitable for CAR T-cell therapy. These conditions amplify the risk of immune effector cell-associated neurotoxicity syndrome (ICANS), which may worsen significantly with treatment [200]. Furthermore, active or poorly controlled autoimmune diseases unrelated to the targeted condition increase the likelihood of severe immune-related complications, making these patients ineligible [202]. Patients with severe lymphopenia (absolute lymphocyte count <500 cells/μL) or significant bone marrow suppression (absolute neutrophil count <1000 cells/μL) face challenges in CAR T-cell manufacturing and recovery, further limiting their eligibility [203]. Lastly, women who are pregnant or breastfeeding are excluded due to the unknown risks of therapy on fetal development and lactation, underscoring the importance of pre-treatment screening to ensure patient safety.

### 10.2 B) adverse events In car T-cell therapy and their management

• **Cytokine Release Syndrome (CRS)**

CRS is the most common adverse event observed in CAR T-cell therapy, caused by excessive immune activation and the release of pro-inflammatory cytokines like IL-6, IL-1, and TNF-α [197]. It typically manifests within the first week post-infusion, presenting as fever, hypotension, tachycardia, hypoxia, and in severe cases, multiorgan dysfunction [200]. The ASTCT grading system is used to assess the severity of CRS and guide treatment [201].

For Grade 1 CRS, supportive care with antipyretics and intravenous fluids is sufficient. In Grade 2 cases, where hypotension requires vasopressors or oxygen support is needed, tocilizumab, an IL-6 receptor antagonist, is the first-line treatment [197]. Tocilizumab is administered at 8 mg/kg (maximum 800 mg/dose), and the dose can be repeated every 8 h up to a maximum of four doses. For refractory CRS or higher grades (3–4), corticosteroids such as dexamethasone (10 mg every 6 h) are added to suppress excessive inflammation. Monitoring of biomarkers like ferritin, CRP, and IL-6 levels aids in early detection and tracking the response to therapy.

• **Neurotoxicity (ICANS)**

Immune effector cell-associated neurotoxicity syndrome (ICANS) is another frequent complication, often occurring alongside or following CRS. Symptoms range from mild (headache, confusion) to severe (seizures, cerebral edema, and coma) [197]. Early recognition using the ASTCT ICANS grading system is essential for effective management.

Treatment for ICANS depends on severity. Grade 1 cases are monitored closely without specific interventions. For Grade 2 neurotoxicity, corticosteroids, typically dexamethasone (10 mg every 6 h), are initiated immediately. In more severe cases (Grade 3–4), methylprednisolone (1–2 mg/kg/day) is preferred. Seizures are managed with antiepileptic drugs such as levetiracetam, while osmotherapy (like mannitol or hypertonic saline) is indicated in cases of cerebral edema [197]. Unlike CRS, tocilizumab is not effective for neurotoxicity, reinforcing the need for prompt corticosteroid use. Continuous neurological assessments and, if necessary, imaging such as MRI help evaluate and manage severe cases [200].

• **Infections and Prophylactic Strategies**

Infections are a critical concern during CAR T-cell therapy due to prolonged immunosuppression and treatment-related cytopenias. The most common infections within the first month are bacterial, caused by gram-positive and gram-negative organisms, often linked to neutropenia and mucosal barrier injury [200]. As the treatment progresses, opportunistic fungal infections and viral reactivations, including herpes simplex virus (HSV) and cytomegalovirus (CMV), become significant risks [201].

Prophylactic antimicrobials are essential to prevent these complications. Trimethoprim-sulfamethoxazole is recommended to prevent bacterial infections and *Pneumocystis jirovecii* pneumonia (PJP). Antiviral prophylaxis with acyclovir or valacyclovir should be initiated to prevent herpesvirus reactivation. For patients at risk of fungal infections, especially those with prolonged neutropenia or receiving high-dose corticosteroids, antifungal prophylaxis with fluconazole or voriconazole is advised [197]. Prophylaxis should ideally start before CAR T-cell infusion and continue for at least six months post-therapy or until immune recovery. For high-risk patients, CMV DNA should be monitored regularly to enable preemptive antiviral treatment.

• **Long-Term Monitoring and Risks**

Long-term follow-up after CAR T-cell therapy is crucial to monitor for delayed adverse effects, including secondary malignancies and late-onset infections. CAR T-cell persistence should be assessed through flow cytometry or qPCR to ensure sustained therapeutic effects [203]. Immunoglobulin levels should be checked every three to six months, and intravenous immunoglobulin (IVIG) replacement initiated for levels below 400 mg/dL to prevent recurrent infections.

Secondary malignancies, although rare, can result from insertional mutagenesis, especially in lentiviral CAR constructs [[197], [204]]. Regular monitoring using TCR repertoire sequencing or peripheral blood genomic analysis is essential to detect clonal expansions. In autoimmune indications, disease-specific biomarkers such as anti-dsDNA for SLE should be tracked to identify potential relapses [202].

• **Quality of Life and Psychological Support**

The physical and psychological burden of CAR T-cell therapy necessitates a multidisciplinary approach to patient care. Addressing therapy-related stress, depression, and anxiety should be an integral part of long-term follow-up. Regular assessments of patient-reported outcomes and quality of life metrics can help identify areas requiring intervention. Access to psychological counseling and support groups is particularly valuable for patients navigating the challenges of treatment and recovery [[200], [205]].

## 11 Discussion

Cell therapy has emerged as a transformative approach in cancer treatment, particularly for hematological malignancies [[10], [11], [12], [13], [14]], and is now gaining traction as a potential therapeutic strategy for autoimmune diseases [206]. Unlike traditional therapies, which primarily aim at suppressing the immune response, CAR T-cells provide a more targeted and durable intervention by eliminating autoreactive immune cells. Recent advancements in CAR T-cell therapies targeting B cells, especially those directed at CD19 and BCMA, have demonstrated significant promise in managing autoimmune conditions such as SLE, MS, and PV [1,[20], [21], [22],[26], [27], [28], [29]].

The clinical benefits of CAR T-cell therapy in these diseases lie in its ability to target and deplete autoreactive B cells, which play a central role in their pathogenesis. The precision of CAR T-cells allows them to deeply penetrate tissues, thereby eliminating autoreactive B cells at their source, a key advantage over traditional B-cell-depleting therapies like rituximab, which often fails to target tissue-resident B cells. This capability is particularly relevant in severe autoimmune manifestations, such as lupus nephritis [165] and neuropsychiatric lupus [166], where autoreactive B cells contribute to substantial tissue damage [168,169].

Although the early results of CAR T-cell therapy in autoimmune diseases are promising, significant challenges remain. CRS and neurotoxicity, while typically milder in autoimmune patients than in cancer patients, still present considerable risks [31,32]. Furthermore, patient responses to CAR T-cell therapy can be highly variable, particularly in those with refractory or severe disease [22]. These variations highlight the need for continued refinement of CAR T-cell manufacturing and administration protocols.

A critical area for further investigation is the long-term persistence of responses following CAR T-cell therapy. Initial clinical data in SLE and PV demonstrate that remission may be sustained despite B-cell reconstitution. However, it remains unclear whether this reflects a durable immune reset or the potential for reemergence of autoreactive B-cell clones. Recent findings from a CD19 CAR T-cell study in autoimmune diseases observed B-cell reconstitution after a median of 112 days, with remission persisting for up to 29 months [174]. Notably, autoantibodies remained undetectable, and reconstituted B cells predominantly exhibited a naive phenotype [174]. This suggests that CAR T-cell therapy may induce a profound immune reset rather than simple B-cell depletion. Long-term follow-up and larger cohort studies are essential to confirm these observations.

Another promising development is the application of CAAR T-cells, particularly in diseases such as PV [183,184]. These T-cells are designed to target pathogenic B cells expressing disease-specific autoantibodies, offering a highly targeted approach with minimal off-target effects. Early-phase trials of CAAR T-cells in PV have demonstrated favorable results, but larger studies are needed to validate these findings and ensure the safety and efficacy of this therapy in broader patient populations [184].

Additionally, in diseases where T cells play a more prominent pathogenic role, such as MS and T1D, CAR Treg therapies are being explored. These therapies aim to reprogram Teff into Tregs, which can modulate the immune response and restore tolerance [192]. Early preclinical data suggest that CAR Tregs may prevent disease progression in MS and T1D [192,207], but further research is required to optimize these approaches and determine their long-term efficacy.

In conclusion, while CAR T-cell therapy offers substantial promise for the treatment of autoimmune diseases, several critical challenges remain. Long-term safety, optimal patient selection, and strategies for preventing relapse are key areas that require further investigation. With ongoing research and clinical trials, CAR T-cell therapy has the potential to become a standard therapeutic option for complex autoimmune diseases. The continued evolution of precision medicine and cellular therapies will be crucial in unlocking the full potential of CAR T-cell therapy for autoimmune disease management.

## Funding

Our laboratory receives funding from the Paula & Rodger Family Foundation and the Michelson Foundation. These funds were utilized to support the publication of this article.

## CRediT authorship contribution statement

**Pedro Franco-Fuquen:** Writing – review & editing, Writing – original draft. **Juana Figueroa-Aguirre:** Writing – review & editing, Writing – original draft. **David A. Martínez:** Writing – review & editing, Writing – original draft. **Eider F. Moreno-Cortes:** Writing – review & editing, Writing – original draft. **Juan E. Garcia-Robledo:** Writing – review & editing, Writing – original draft. **Fabio Vargas-Cely:** Writing – review & editing, Writing – original draft. **Daniela A. Castro-Martínez:** Writing – original draft. **Mustafa Almaini:** Supervision. **Januario E. Castro:** Writing – review & editing, Supervision.

## Declaration of competing interest

The authors declare that they have no known competing financial interests or personal relationships that could have appeared to influence the work reported in this paper.

## Data Availability

No data was used for the research described in the article.
